# Investigations of New Phenothiazine-Based Compounds for Dye-Sensitized Solar Cells with Theoretical Insight

**DOI:** 10.3390/ma13102292

**Published:** 2020-05-15

**Authors:** Aneta Slodek, Dawid Zych, Grażyna Szafraniec-Gorol, Paweł Gnida, Marharyta Vasylieva, Ewa Schab-Balcerzak

**Affiliations:** 1 Institute of Chemistry, University of Silesia, Szkolna 9, 40-006 Katowice, Poland; grazyna.szafraniec-gorol@us.edu.pl; 2 Independent Researcher, Poland; dawidzych92@gmail.com; 3 Centre of Polymer and Carbon Materials, Polish Academy of Sciences, M. Curie-Sklodowska 34, 41-819 Zabrze, Poland; pgnida@cmpw-pan.edu.pl (P.G.); mvasylieva@cmpw-pan.edu.pl (M.V.)

**Keywords:** photovoltaic, phenothiazine, donor-acceptor system, photophysical studies, DFT/TD-DFT calculations

## Abstract

New D-π-D-π-A low-molecular-weight compounds, based on a phenothiazine scaffold linked via an acetylene unit with various donor moiety and cyanoacrylic acid anchoring groups, respectively, were successfully synthesized. The prepared phenothiazine dyes were entirely characterized using nuclear magnetic resonance (NMR) spectroscopy and elemental analysis. The compounds were designed to study the relationship between end-capping donor groups’ structure on their optoelectronic and thermal properties as well as the dye-sensitized solar cells’ performance. The effect of π-conjugation enlargement by incorporation of different heterocyclic substituents possessing various electron–donor affinities was systematically experimentally and theoretically examined. Density functional theory (DFT) and time-dependent density functional theory (TD-DFT) calculations were implemented to determine the electronic properties of the novel molecules.

## 1. Introduction

Currently, dye-sensitized solar cells (DSSCs) based on metal-free organic dyes have drawn greater and greater attention owing to their advantages, such as a relatively low cost, compliance with environmental requirements, and easy device preparation. Opposite to the complexes with heavy atoms utilized in DSSCs, which show high or even comparable power conversion efficiency (PCE) of 13% [1,2,3,4,5], the use of metal-free sensitizers in DSSCs makes it possible to obtain ever-growing PCE up to 14% [6,7,8,9,10]. The synthesis of organic dyes such as carbazole, thiophene, pyrene, phenothiazine to act as donors (D), and quinoline and pyridine as acceptor (A) units, produce in combination D-A systems which have been evolved for the utilization of DSSCs, organic light-emitting diodes (OLEDs), sensors and bio-imaging [7,8,9,10,11,12,13,14,15,16,17,18,19,20,21,22,23]. Phenothiazine (PTZ), as a heterocyclic compound, encloses electron-abundant sulfur and nitrogen atoms, and plays the role of an strong electron-donating (D) moiety in D-A systems. The large π-conjugated structure, high molar absorption coefficients (ε), high thermal and chemical stability, and also the non-planar conformation preventing aggregation of phenothiazine are important requirements making PTZ and its derivatives desirable candidates for photovoltaic applications. The many ways to modify phenothiazine structure by addition of different types of substituents and/or elongation of π-conjugation with various linkers, which make it possible to produce dyes with desirable optoelectronic properties and significant photovoltaic performance [7,17,23,24,25,26]. With hexylphenothiazine attached to porphyrins (G10 and G11) exploited as a sensitizer, improved efficiency of 5.1–5.4% was reported. The optical and electrochemical characterization of dyes G10 and G11 implied that a photoinduced intramolecular electron transfer mechanism took place from hexylphenothiazine (D) to acetic acid (A) through the porphyrin macrocycle. Thus, structural modifications of different electron donors and acceptors substituted with porphyrin sensitizers promote efficient and stable DSSCs for better light-harvesting and efficient operation under ambient light conditions [7]. Four asymmetric D-π-A dyes based on double donors, such as phenothiazine and carbazole connected through the butyl or octyl chain, with the electron acceptor being cyanoacetic acid or rhodanine acetic acid, have exhibited a large molar extinction coefficient and beneficial light-harvesting capability of the DSSCs. The higher open-circuit voltage (V_OC_) for dyes with octyl chains compared to those with butyl chains was observed. The dyes containing octyl chains favor anti-aggregation effects and low charge recombination rate and exhibit the highest short-circuit photocurrent density (9.64 mA cm^−2^) and open-circuit photovoltage (720 mV), causing the best overall power conversion efficiency of 4.76% [26]. The phenothiazine dyes with dithieno[3,2-b:2′,3′-d]pyrrole (DTP) as terminal groups were reported to study the structure–activity relationship of phenothiazine organic dyes. The different conjugation order of PTZ and DTP, as well as the substituent group on the DTP unit, play a fundamental role in the light-harvesting capacity, the highest occupied molecular orbital/the lowest unoccupied molecular orbital (HOMO/LUMO) energy levels, interfacial charge transfer and recombination in solar cell devices with PCE from 5.70% to 7.70% [8]. The effects of variation in the substitution geometry (at the 2, 2,7 and 3,8 positions) and π-spacers (the thiophene and phenyl) in phenothiazine dyes on photovoltaic performance were investigated [25]. The overall performance of compounds substituted at the 2,7 and 3,8 positions was nearly identical, although the compounds with 3,8 geometry exhibited a 10% higher dye loading without additives. The most efficient device had the PTZ substituted at the 3,8-positions with a *p*-methoxyphenyl auxiliary donor and thiophene π-linker, giving a PCE of 5.88% with respect to the reference N719-based dye (7.92%). The effect of the donor groups of oxindole-phenothiazine sensitizers (POTZP1-POTZP3) on the photovoltaic performance was reported. The introduction of an additional phenothiazine unit in POTZP2 gave the enhancement of short-circuit photocurrent density (Jsc) values, broader absorption spectra, and higher conversion efficiency (5.91%) compared to the reference dye containing one PTZ group, with PCE being 3.97% [24]. It has been confirmed that the three-anchoring dye consists of the addition of extra donor units as well as a linker between the phenothiazine, with cyanoacrylic acid (D-A) units linked by triarylamine resulting in higher molar extinction coefficients in the intramolecular charge transfer (ICT) bands, less dye aggregation on the TiO_2_ surface, and better DSSC performance than mono-anchoring dye, where the best cell efficiency, 7.06%, was achieved for the dye with two additional donor units [27].

Recently, we presented D/A-π-PTZ-π-A dyes based on a PTZ framework, intending to study the impact of the auxiliary donor *p*-methoxyphenyl and acceptor 3,5-bis(trifluoromethyl)phenyl) moieties of dyes on photophysical and thermal properties and photovoltaic performance [17]. We have shown that a DSSC device based on a sensitizer with an electron-acceptor group confirmed a significantly lower PCE, of 5.03%, than one with an electron-donor unit (PCE = 6.21%), with respect to the reference N719-based dye (3.56%).

Thus, in this contribution, we designed and synthesized three new novel dyes based on phenothiazine scaffold 2a–2c of D-π-PTZ-π-A architecture with diverse type end-capping donor moieties and a cyanoacrylic group acting as an acceptor connected via acetylene spacer. According to previously reported papers, the presence of an extra donor end-capped unit (D) in systems D-π-D-π-A has improved the DSSC performance relative to the D-π-A and/or A-π-D-π-A analogues in terms of charge recombination, long-term stability, and photocurrent increasing [17,28,29,30]. We evaluated the selection of three donor end-capped groups, dibenzothiophene (2a), bithienyl (2b), and 9,9’-dibutylfluorenyl (2c), to study the relationship between their structure and properties and the influence of the electron-donating ability of selected donors on tuning the photovoltaic parameters of dyes 2a–2c. The photophysical and photovoltaic properties of novel 2a–2c dyes were thoroughly investigated and confronted with DFT and TD/DFT calculations.

## 2. Materials and Methods

All chemicals and starting materials were purchased from commercial sources. All reactions were performed under argon atmosphere. Column chromatography was carried out on Merck silica gel. Thin-layer chromatography (TLC) was performed on silica gel (Merck TLC Silica Gel 60). Fluorine doped tin oxide coated glass slides (FTOs, 7 Ω/sq, Sigma-Aldrich, St. Louis, MO 63103, USA), 18NR-T Titania Paste (Greatcell Solar Materials, Queanbeyan East NSW 2620, AUS), surfactant (Hellmanex III, Hellma Analytics, Plainview, NY, USA), isopropanol (IPA, POCH, Gliwice, Poland), di-tetrabutylammonium cis-bis(isothiocyanato)bis(2,2′-bipyridyl-4,4′-dicarboxylato)ruthenium(II) (NCS)_2_ denoted as N719 and EL-HSE electrolyte were purchased from Sigma-Aldrich.

The NMR spectra were recorded on a Bruker Avance 400 MHz instrument and Avance II 600 MHz Ultrashield Plus (Bruker, Warszawa, Poland) by using CDCl_3_ and DMSO-d_6_ as solvents.

Elemental analysis was registered by Vario EL III apparatus (Elementar, Langenselbold, Germany).

High-resolution mass spectrometry (HRMS) measurements were performed by using Mass Spectrometer type Q-TOF maXis impact (Bruker Daltonics, Warszawa, Poland). The samples were weighed and dissolved in chloroform to give a sample concentration of 1 mg mL^−1^. The samples were then diluted 100 times with solution water: acetonitrile 10:90 (*v*/*v*) with the addition of 0.1% formic acid.

Differential Scanning Calorimetry (DSC) was performed by TA-DSC 2010 apparatus (TA instruments New Castle, Germany), under a nitrogen atmosphere with a heating/cooling rate of 20 ºC/min and using aluminium sample pans.

The absorptions spectra in the UV-Vis range of dyes solutions and adsorbed onto TiO_2_ substrate were registered in the range of 200–1100 nm using a V-570 UV-Vis-NIR Spectrophotometer (Jasco Inc. Easton, MD, USA).

The morphology of the surface of electrodes in nanoscale was characterized by atomic force microscopy (AFM) using TopoMetrix Explorer device (Industriële Veiling Eindhoven B.V., CA Eindhoven, the Netherlands), operating in contact mode, in air, in constant force regime.

The photovoltaic performance was determined using a PV Solutions Solar Simulator (Sciencetech Inc. London, Ontario, Canada) and a Keithley 2400 (Tektronix, Inc., Beaverton, OR, USA). The IPCE spectra were registered by a photoelectric spectrometer (Photonic Institute, 31-267 Kraków, Poland).

The electrochemical cell comprised of the platinum electrode with a 1 mm diameter of Pt as a working electrode, an Ag|Ag^+^ electrode as a pseudo-reference electrode and a platinum coil as an auxiliary electrode. Measurements were conducted at room temperature at a potential rate of 50 mV/s and were calibrated against a ferrocene/ferrocenium redox couple. Cyclic voltammetry measurements were conducted in 1.0 mM concentrations of all compounds. Electrochemical studies were undertaken in 0.1 M solutions of Bu_4_NBF_4_, 99% (Sigma-Aldrich) in dichloromethane (DCM) CHROMASOLV ^®^, 99.9% (Sigma-Aldrich).

The FTOs were washed in a mixture of deionised water with Hellamanex (9:1 by vol.) for 5 min and then rinsed twice by hot distilled water (50 mL). Such slides were ultrasonicated in isopropyl alcohol for 5 min and rinsed twice by hot distilled water (50 mL). After washing FTOs were dried in hot air. The layer of TiO_2_ was screen-printed on cleaned FTO and was dried at 125 °C for 5 min and then other layers were printed. Next, the coated FTOs were fired at 500 °C in the air for 30 min. Platinum-based counter electrodes were prepared also by screen printing method following by thermal treatment at 500 °C for 30 min.

The TiO_2_ substrates were immersed in dye-solution for dyes 2a, 2b and 2c (3 × 10^−4^ M in CHCl_3_) and for commercial dye (N719) it was the same concentration (3 × 10^−4^ M) but in MeOH. After 24 h, excess of a dye was flush by MeOH. The next step was drying the TiO_2_ electrodes in the air. Such photoanodes with adsorbed dye’s molecules were employed to assembly a sandwich-structure solar cells (FTO/TiO_2_+dye/EL-HSE/Pt/FTO) by fixing it with the counter electrode (Pt/FTO). The electrolyte consist of iodide/triiodide redox couple was injected between the electrodes. The photovoltaic performance of fabricated devices was registered under standard 100 mW/cm^2^ (AM 1.5 G) irradiation.

## 3. Results and Discussion

### 3.1. Synthesis and Characterization

**Scheme 1:**
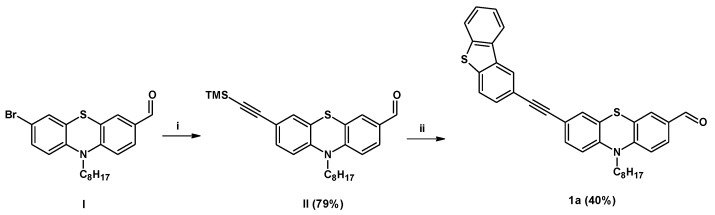
Synthetic route for the preparation of compounds **1a**. Reagents and conditions: (i) TMSA, [PdCl_2_(PPh_3_)_2_]/PPh_3_/CuI, piperidine, reflux, 4 h; (ii) 2-bromodibenzothiophene, [Pd(PPh_3_)_4_]/PPh_3_/CuI, TBAF, TEA/THF, 60 °C, 24 h.

The novel phenothiazine derivatives (2a–2c) of D-π-D-π-A type with various donor end-capped 2,2′-bithienyl, dibenzothiophenyl, and 9,9’-dibutylfluorenyl moieties linked by ethylene with phenothiazine were synthesized by multi-step reactions. First, the 7-bromo-10-octyl-10*H*-phenothiazine-3-carbaldehyde (I) obtained in multistep reactions according to well-known synthetic procedures was used as a starting material in the synthesis of compound 1a (Scheme 1) [23,31,32]. Compound I was converted to 2-[(trimethylsilyl)ethynyl]-10-octyl-10*H*-phenothiazin-3-carbaldehyde (II) via [Pd]-catalyzed cross-coupling reaction using TMSA (79% yield). Subsequently, prior deprotection of II using tetrabutylammonium fluoride (TBAF) followed by in-situ [Pd]-catalyzed reaction with 2-bromodibenzothiophene gave compound 1a a 40% yield.

**Scheme 2:**
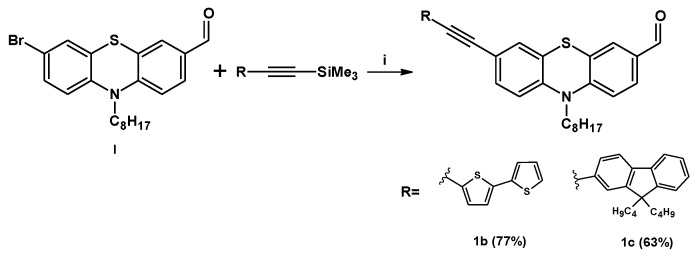
Synthetic route for the preparation of compounds 1b and 1c. Reagents and conditions: (i) [Pd(PPh_3_)_4_]/PPh_3_/CuI, TBAF, THF, 65 °C, 24 h.

**Scheme 3:**
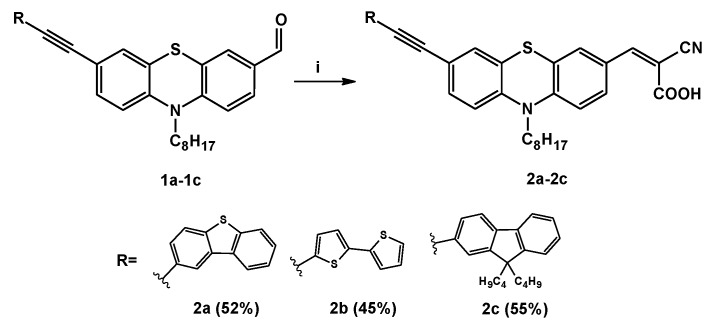
Synthetic route for the preparation of compounds 2a–2c. Reagents and conditions: (i) cyanoacrylic acid, piperidine, CHCl_3_, reflux, 24 h.

The compounds 1b and 1c were obtained by Sonogashira cross-coupling of I with previously synthesized 4-[(trimethylsilyl)ethynyl]arenes (Scheme 2) [33,34]. Finally, the broadly used Knoevenagel condensation of 1a–1c with cyanoacetic acid (Scheme 3) gave the desired compounds 2a–2c average yields of 45–55%.

The compounds were fully characterized by the NMR spectroscopic technique (Supporting Information, Figures S1–S12). The purity of prepared molecules was confirmed by mass spectrometry and elemental analysis (Supporting Information, Synthesis and characterization). In the ^1^H NMR spectra for all compounds 2a–2c, the characteristic signal for a proton from the methylene group appeared as a singlet at 8.00 ppm. Apparently, in the proton NMR spectra of compounds 2a–2c, the signal of a carbaldehyde proton (with a chemical shift at 10 ppm), which is characteristic for aldehyde derivatives 1a–1c, disappeared. Additionally, comparing the proton NMR spectra of aldehydes 1b and 1c with the target compounds 2b and 2c, the signals from the proton at the C4 phenothiazine ring were moved to higher chemical shifts, at about 0.15 ppm. In the ^13^C NMR spectra of 2a–2c dyes, the lack of peaks typical for carbons from a carbaldehyde group at about 190 ppm, and the appearance of a signal at about 165 ppm, which is the standard signal for the carbon of carboxyl groups (-COOH), was observed. Further, in the carbon NMR spectra of 2a–2c, peaks for methylene carbon (-CH=C-) at about 100 ppm were seen.

The target phenothiazine derivatives 2a–2c were obtained as crystalline compounds from reaction with melting temperature (T_m_), identified by DSC during the first heating scan, in the range of 56–68 °C (Experimental section in Supporting Information and Figure S13). The observed broad-melting endotherm proves a low degree of crystallinity of these molecules, probably due to defects, which cause partial disruption of the crystalline order, decreasing the crystallinity [35]. Defects such as crystal imperfections, which are defects in the regular geometrical arrangement of the atoms in a crystalline solid, may result from the crystallization process. On the other hand, impurities can also affect the broad peak obtained during the melting process. In the case of presented compounds, the purity was confirmed by elemental analysis. The differences in calculated and found C, N and H content are in the acceptable range, not exceeding 0.3% (Experimental Section in Supporting Information). However, it should be kept in mind that even a trace amount of impurities can impact the DSC curve trace. When the isotropic liquid was cooled down and heated again, the glass transition (T_g_) phenomena was seen, with T_g_ ranging from 43 to 64 °C (Figure S13). Thus, the synthesized compounds are molecular glasses which can form glassy phases above room temperature and uniform amorphous thin films can be prepared. Considering the DSC thermograms of 2a–2c, it can be concluded that they form stable amorphous molecular materials because on further heating above their T_g_ no peaks due to crystallization and melting were seen (Figure S13). It was found that introduction to phenothiazine-derivative dibenzothiophenyl unit (2a) increased both T_m_ and T_g_ compared to other substituents, that is, 2,2′-bithienyl (2b) and 9,9′-dibutylfluorenyl (2c). Summarizing, the synthesized phenothiazine derivatives were characterized by a low degree of crystallinity, confirmed by broad-melting endotherm with low enthalpy and showed a significant tendency for the creation of amorphous material.

### 3.2. Electrochemical Properties

In the case of compounds tested as dyes in DSSCs, the electrochemical properties are crucial considering the electron injection and dye regeneration process, which take place in the device during the conversion of light on electricity. Two techniques, cyclic voltammetry (CV) and differential pulse voltammetry (DPV), were used for characterizing the electrochemical behaviour of 2a–2c. Results of these investigations are presented in Table 1. The highest occupied molecular orbitals and the lowest unoccupied molecular orbitals (LUMO) of compounds were calculated using the electrochemical oxidation and reduction onset potentials, respectively.

**Figure 1:**
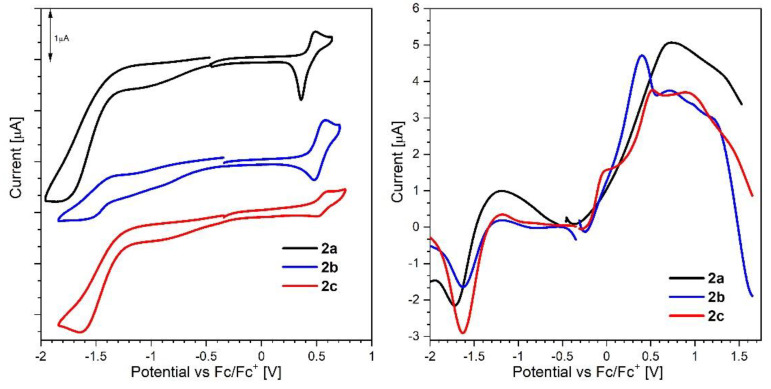
CV and DPV curves of 2a–2c in a dichloromethane solution. The measurements were performed with a platinum working electrode and referenced against an Fc/Fc^+^ couple, scanning rate 50 mV/s.

Compounds 2a–2c were characterized by a non-reversible reduction process. For phenothiazine derivatives, the onset of the reduction peak was registered at the potential of −1.34 V vs. Fc/Fc^+^ for 2b to −1.22 V vs. Fc/Fc^+^ for 2c (Figure 1). Reduction potentials correspond to the reduction of cyanoacrylic acid [36]. However, taking into account the structure, not only the acid unit but also the PTZ moiety takes part [17].

The oxidation process is important for compounds tested as dyes in DSSCs. The compounds should exhibit similar potential to the potential of the pair I_3_^−^/I^−^ being 0.42 V, enabling the regeneration of dyes by I^−^ in the electrolyte [37]. Compound 2a was characterized by a non-reversible oxidation process. After three scans, no product of electrode surface was found (Figure S14). Based on the peak-to-peak separation (cathodic/anodic waves) (Table S1) oxidation processes for 2b and 2c were quasi-reversible.

Oxidation to potentials higher than first oxidation potentials cause an irreversible process. The oxidation process accords to the oxidation of the PTZ unit [38]. The lowest oxidation potential of 0.40 V was observed for 2a and the highest, 0.47 V, for 2c. These values characterized the electron-donating nature of the PTZ part. Differences in oxidation potentials can be caused by the donating effect of linked units. The highest influence on phenothiazine dyes has bithiophene in 2b, but this effect is notably smaller than in the case of the 3,5-bis(trifluoromethyl)phenyl and *p*-methoxyphenyl moieties previously reported [17].

On the other hand, for efficient electron injection, the energy state level (LUMO) should be above that of the conductivity band of TiO_2_ (4.0 eV) [39]. Considering the electrochemical data, it can be concluded that the synthesized new phenothiazine derivatives show the existence of a driving force for electron injection into the TiO_2_ conductivity band and regeneration of dyes in the electrolyte based on an iodine redox pair.

Energy gaps were determined using two methods, that is, electrochemical measurements and UV-Vis electronic spectra registered in dichloromethane. It was found that the presence of 9,9′-dibutylfluorene substituent in 2c reduced the value of E_g_^CV^ to 1.69 eV in comparison with molecules containing dibenzothiophene (2a) and 2,2′-bithiophene (2b) units.

### 3.3. Structure Optimization and Frontier Molecular Orbitals

The structures of the target compounds were optimized by using the method based on density functional theory (DFT) and also with the time-dependent extension (TD-DFT). DFT calculations were performed in the program Gaussian 09, with the B3LYP [40] exchange-correlation functional with the 6-31G** basis set, whereas in the case of TD-DFT, the CAM-B3LYP [41] and wB97XD [42] exchange-correlation functionals with the 6-31G** basis set were applied [43]. All calculations were performed in a chloroform solution in the polarizable continuous model (PCM) [44]. The optimized structures of molecules 2a–2c, with the contours and energies of HOMO-1, HOMO, LUMO and LUMO+1, are presented in Table 2.

**Figure 2:**
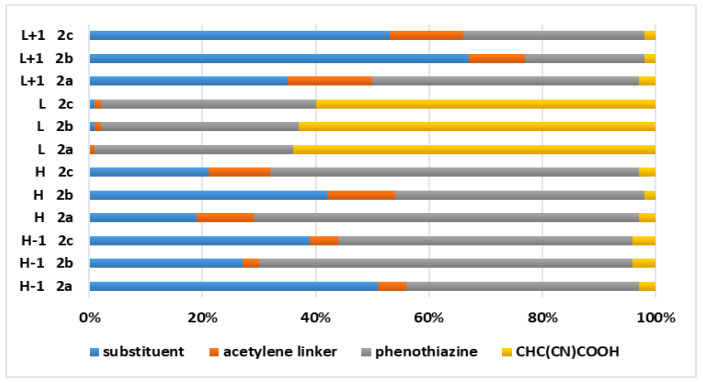
The contributions of particular parts of molecules 2a–2c to the creation of selected orbitals.

The highest value of energy gap ΔE = 2.79 eV was achieved by 2a, followed by 2c (ΔE = 2.76 eV) and 2b (ΔE = 2.63 eV). The percentage contribution of particular parts of the molecules 2a–2c in the creations of selected orbitals is presented in Figure 2.

Due to the various electron-donating characters of the substituents, i.e., 2,2′-bithienyl, dibenzothiophenyl, and 9,9′-dibutylfluorenyl, the contributions to the creation of HOMO differ from each other; the highest (42%) was observed for 2,2′-bithienyl (2b) and the lowest (19%) for dibenzothiophenyl (2a) group. The lead structure, 10-octyl-10*H*-phenothiazine, has a dominant contribution to the creation of the highest molecular orbital (44–68%), whereas the difference of acetylene linker and anchoring group (-CHC(CN)COOH) between the studied compounds is not significant. Moreover, a deep analysis of the optimized structures of 2a–2c revealed that substituents are coplanar in relation to the wing of the butterfly structure of phenothiazine.

**Figure 3:**
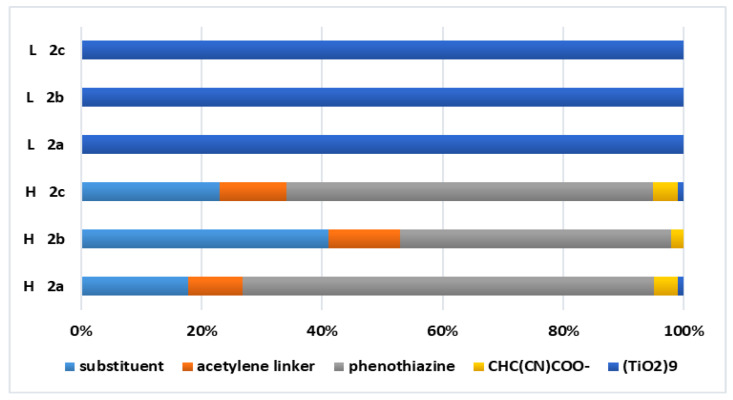
The contributions of particular parts of systems 2a–2c/(TiO_2_)_9_ to the creation of frontier orbitals.

Considering the fact that the prepared phenothiazine derivatives will be tested as a sensitizer in DSSCs, the theoretical calculations of systems-type dye/TiO_2_ (based on chemisorption model) were realized at the B3LYP/6-31G(d,p) level of theory, with the LANL2DZ basis set for Ti atoms with implemented titanium clusters (TiO_2_)_9_ [17]. The optimized structure of systems 2a–2c/(TiO_2_)_9_ are presented in Table 3. The same trend of the localization of the highest occupied molecular orbital for studied systems 2a–2c/(TiO_2_)_9_, as for the dyes 2a–2c, was followed. LUMOs are located only on the (TiO_2_)_9_ cluster (Figure 3). Calculated energy gaps for 2a/(TiO_2_)_9_ (1.95 eV) are the highest, whereas the lowest was achieved for 2b/(TiO_2_)_9_ (1.80 eV). The optimized geometries did not demonstrate any noteworthy differences between the optimized dyes and their corresponding dye–TiO_2_ systems.

### 3.4. Experimental and Theoretical Optical Properties

**Figure 4:**
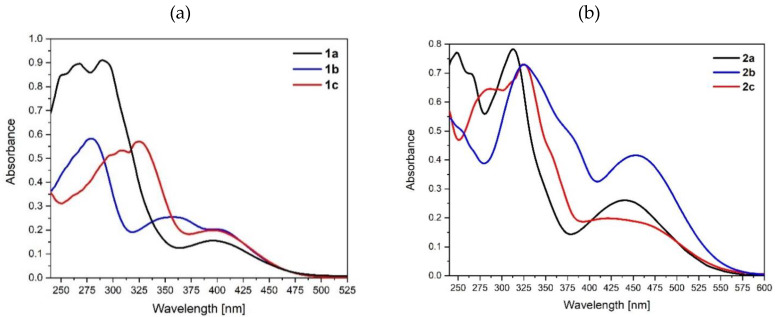
(**a**) Absorption spectra of 1a–1c and (**b**) 2a–2c in a chloroform solution (c = 10^−5^ mol L^−1^).

The UV-Vis spectra of precursors 1a–1c and target dyes 2a–2c in chloroform solution are presented in Figure 4, and the optical data are summarized in Table 4. The precursors 1a–1c display several absorption peaks in the UV region, ascribed to π-π* transition and the other peaks at 395 nm caused by ICT from the donor to acceptor. The dyes 2a–2c show absorption peaks of π-π* transition at similar wavelengths as precursors 1a–1c, whereas the ICT bands are red-shifted around 50 nm. With regards to the donor terminal unit, the ICT transition peak of 2a–2c is both hyper- and bathochromic shifted with an increase of donor ability of the substituents in the sequence 1c→1a→1b. In general, the replacement of fluorene with thiophene or dibenzothiophene as terminal substituents in the presented compounds facilitates effective ICT and extends the region of light absorption.

**Figure 5:**
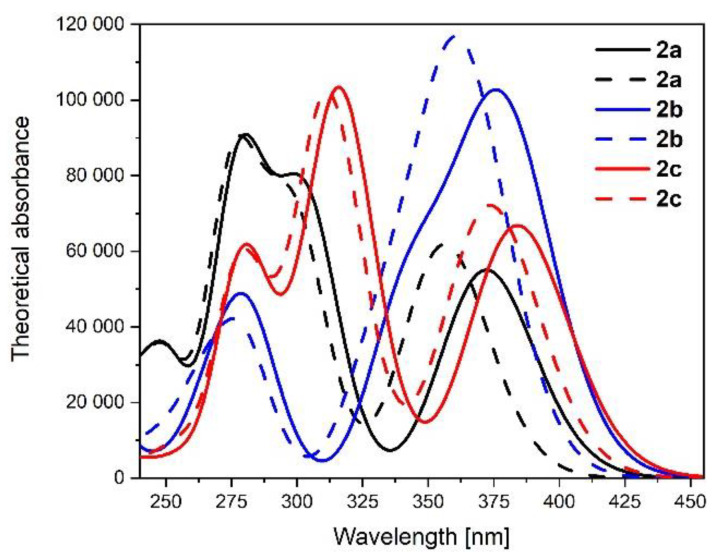
Theoretically calculated CAM-B3LYP (solid line) and wB97XD (dotted line) absorption spectra for 2a–2c.

The theoretical investigations of prediction of the absorption spectra of 2a–2c using CAM-B3LYP and wB97XD have been utilized, and the calculated UV-Vis spectra are presented in Figure 5. The computed and the experimental absorption maxima are in reasonable agreement, differing by about 60 nm.

It can be seen in Figure 5 that the stimulated absorption spectra using both methods CAM-B3LYP and wB97XD follow the same trend, being red-shifted in the direction 2a→2b→2c, differing from the experimental data although the differences in the calculated absorption maxima are precisely small. TD-DFT calculations outcomes appear at least in two excitation wavelengths in the range of 250 to 450 nm with high oscillator strength (f) from 0.76 to 1.51. In particular, compound 2b displays the strongest oscillator strength (f = 1.51) among all the investigated dyes, as well as the smallest band gap of 2.63 eV. In addition, there is a slight difference in the value of the computed band gap among the studied dyes 2a–2c and the optical band gap (Table 2 and Table 4), about 0.4 eV. For all dyes 2a–2c, the theoretical calculations demonstrate that the reasonably strong excitation at 356–384 nm is mainly dominated by a HOMO–LUMO transition, and in some part described by a HOMO-1–LUMO transition (Table 5).

### 3.5. Evaluation of Phenothiazine Derivatives as Sensitizers in DSSCs

The synthesized phenothiazine derivatives were tested in DSSCs. The method of cell preparation is given in the Supporting Information. Devices with sandwich structure FTO/TiO_2_+dye/EL-HSE/Pt/FTO were fabricated. Moreover, the reference solar cell containing commercial dye di-tetrabutylammonium cis-bis(isothiocyanato)bis(2,2′-bipyridyl-4,4′-dicarboxylato) ruthenium(II) (NCS)_2_, denoted as N719, was prepared.

Before focusing on the photovoltaic (PV) performance of prepared cells containing phenothiazine derivatives 2a–2c as sensitizers, it is important to discuss the light-harvesting efficiency (LHE) of 2a–2c and electronic absorption properties with surface morphology of TiO_2_ with anchored dyes. LHE, being an important parameter for the high efficiency of a DSSC, was calculated from the oscillator strength of the dye molecule. The theoretical UV-Vis absorption spectra for 2a–2c obtained using CAM-B3LYP and wB97XD (Figure 5) allowed calculating of LHE. The obtained data are presented in Table 5.

As can be seen from Table 5, higher oscillator strength may enhance the LHE. The synthesized phenothiazine derivatives demonstrate high LHE, which is required for an efficient photocurrent response. The differences in the calculated LHE values of the presented compounds are insignificant.

**Figure 6:**
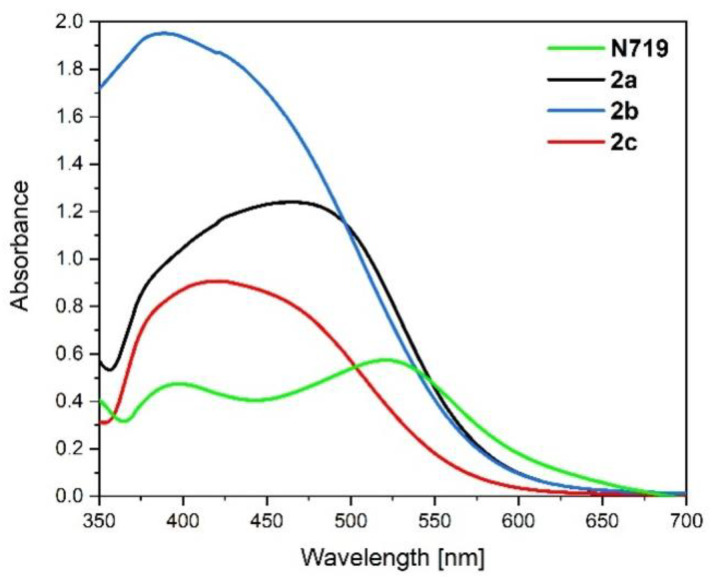
UV-Vis spectra of anchored dyes 2a–2c and N719 on TiO_2_.

Considering the registered UV-Vis spectra of TiO_2_ with phenothiazine derivatives it can be noticed that all such systems exhibited higher light absorption intensity than semiconductor layer with N719 (Figure 6). Thus, it can be expected that devices based on 2a–2c may provide better PV efficiency than a cell with N719.

**Figure 7:**
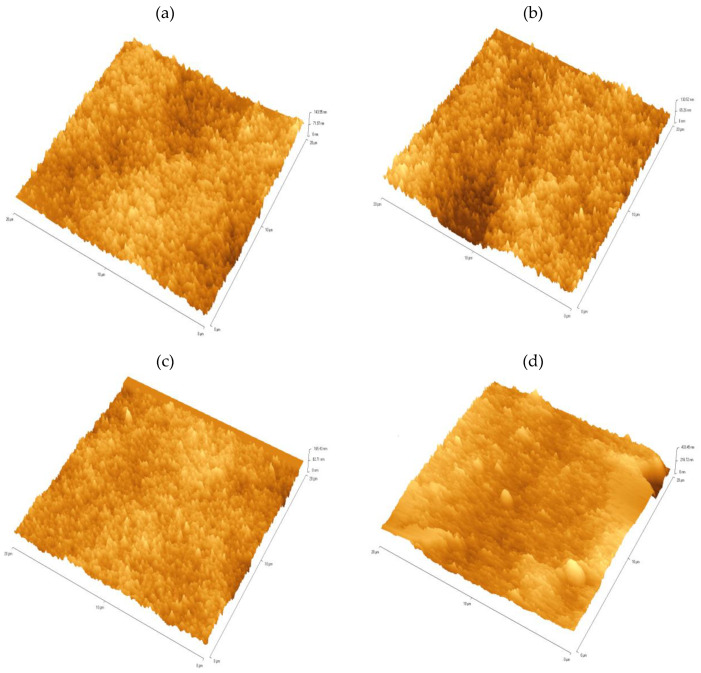
The AFM micrographs of TiO_2_ with anchored (**a**) 2a, (**b**) 2b, (**c**) 2c and (**d**) N719.

The surface morphology of photoanode, which impacts on cell efficiency, was investigated using atomic force microscopy (AFM). The AFM micrographs of the prepared TiO_2_ with adsorbed 2a–2c are presented in Figure 7.

The quality of photoanodes was determined by measuring root-mean-square roughness (RMS). All photoanodes with phenothiazine derivatives were quite planar, as indicated by the low RMS values, which were 18, 17 and 18 nm for 2a, 2b and 2c, respectively. A significant smoothing of the photoanode surface compared to TiO_2_ with adsorbed N719 (RMS = 52 nm) or TiO_2_ without anchored dye (RMS = 86 nm) was observed. This may indicate that the TiO_2_ pores were well filled by the compounds 2a–2c comparing to N719. The lower roughness of photoelectrodes could impact on both the stability of the device and the improvement of PV performance.

**Figure 8:**
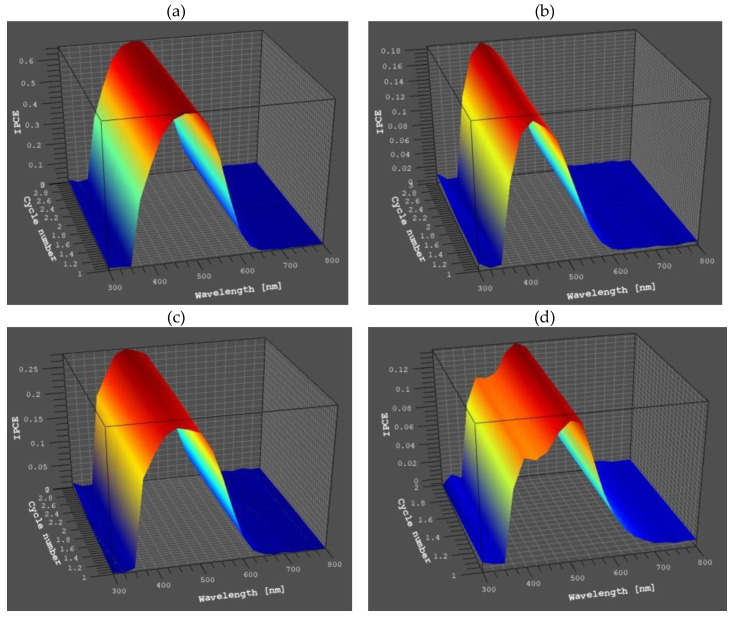
IPCE spectra of prepared devices containing (**a**) 2a, (**b**) 2b, (**c**) 2c and (**d**) N719.

In the next step of investigations, the complete solar cells were fabricated. At the beginning, the incident photon-to-current efficiency (IPCE) of DSSCs was registered. Figure 8 shows the IPCE spectra of constructed DSSCs.

As can be seen from Figure 8 the highest photocurrent response about 60% exhibited the device with phenothiazine containing dibenzothiophenyl unit 2a compare to the cell with 2b (18%) and 2c (27%). Furthermore, all DSSCs based on phenothiazine derivatives showed higher IPCE values than a reference solar cell containing N719 (15%). In the case of a device with compound 2b, the hypochromic shift of the maximum IPCE curve was observed in relation to the other molecules (Figure S15).

**Figure 9:**
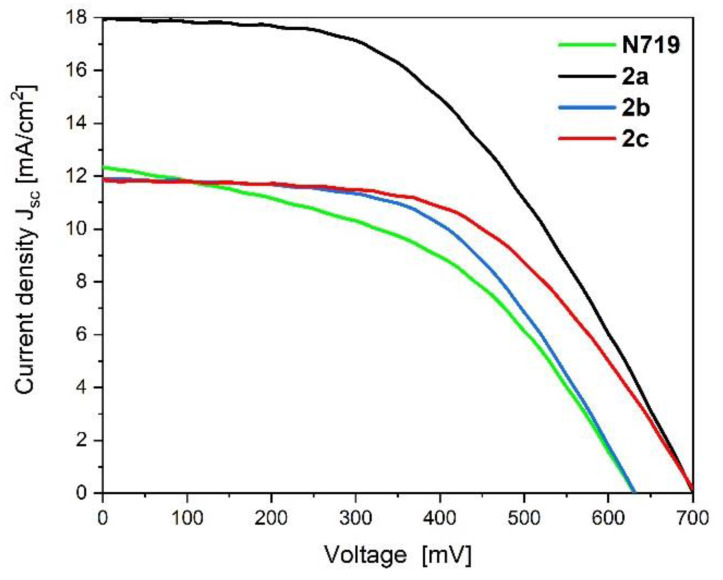
Photocurrent density–voltage (J-V) curves of the prepared devices with dyes 2a–2c.

Finally, the photovoltaic parameters such as open-circuit voltage (V_OC_), short circuit photocurrent density (J_SC_), fill factor (FF) and the power conversion efficiency (PCE) were calculated, based on registered the current density–voltage (J-V) characteristics of prepared solar cells (Figure 9). The PV parameters are collected in Table 6.

As can be seen in Table 6 and Figure 9, all of the tested devices containing phenothiazine derivatives demonstrated better PV performance than the reference solar cell with commercial N719. The DSSC with a compound bearing a dibenzothiophenyl donor moiety 2a exhibited the best conversion efficiency of 6.22% (about 57% of the PV performance of the cell sensitized with N719). The best conversion efficiency of the cell sensitized with 2a results from the improved electron injection and less dark current, as indicated by the fact it had the highest J_SC_ (17.96 mA/cm^2^) and a high V_OC_ (700 mV). The device fabricated using phenothiazines substituted with 2,2′-bithienyl (2b) and 9,9′-dibutylfluorene (2c) showed a lower PCE value than a cell with 2a, probably due to less efficient electron injection into the TiO_2_ conduction band indicated by the lower J_SC_. The obtained results correlate very well with IPCE response (Figure S15). The device based on 2a demonstrated a higher maximum IPCE value than the others, 60%. The IPCE of devices based on 2a and 2c displayed a broader band between 350 and 650 nm than those with 2b. Moreover, DSSC with 2b showed a significantly low intense band, with a maximum IPCE value of 18% compared to 2a and 2c (27%). The better IPCE response can be interpreted in terms of a higher J_sc_ value, which correlates well with the obtained short circuit photocurrent density. It can be concluded that the most promising, as sensitized for DSSC, is 2a, and optimization of the photoanode preparation by changing a solvent or semiconductor layer modification may further enhance the PV parameters.

## 4. Conclusions

In summary, the novel dyes 2a–2c of D-π-D-π-A based on phenothiazine (PTZ) with diverse donor (D) terminal groups connected by ethylene spacer with PTZ, and cyanoacrylic acid as an anchoring group (A) were successfully synthesized and fully characterized. The impact of end-capped groups in dyes 2a–2c on thermal, electrochemical and photophysical properties has been thoroughly studied and widely discussed with DFT/TD-DFT theoretical calculations. It was established that the electron-donating property of the end-capped groups has a moderate influence on the optical absorption properties and HOMO/LUMO energy levels, but a considerable impact on the morphology of the resulting active layers and photovoltaic behaviour of dyes 2a–2c. The DSSC device based on 2a demonstrated an overall PCE of 6.22% (J_sc_ = 17.96 mA/cm^2^, V_oc_ = 700 mV, and FF = 0.48) which was considerably higher than that of 2b (PCE = 4.22%, J_sc_ = 11.87 mA/cm^2^, V_oc_ = 631 mV, FF = 0.54) and 2c (PCE = 4.80%, J_sc_ = 12.80 mA/cm^2^, V_oc_ = 703 mV, FF = 0.56). Moreover, compared to the reference N719-based dye (PCE = 3.56%), the investigated dyes 2a–2c showed appreciably improved photovoltaic performance. These results point out that the terminal donor group in novel dyes 2a–2c plays an important role in the enhancement of the optoelectronic properties and efficiency of DSSC devices.

## Figures and Tables

**Table 1:** Electrochemical data for phenothiazine derivatives 2a–2c measured in 0.1M Bu_4_NPF_6_/CH_2_Cl_2_ solution.

Code	E_ox_^onset^ (V)	E_red_^onset^ (V)	HOMO (eV)	LUMO (eV)	E_g_^CV^ (eV)	E_g_^DPV^ (eV)
**2a**	0.40	−1.32	−5.50	−3.78	1.72	1.76
**2b**	0.42	−1.34	−5.52	−3.76	1.76	1.68
**2c**	0.47	−1.22	−5.57	−3.88	1.69	1.68
IP = −5.1 − E_ox_^onset^; EA = −5.1 − E_red_^onset^; Eg^CV^ = E_ox_^onset^ − E_red_^onset^						

**Table 2:** The optimized structures (6-31G(d,p)/B3LYP), contours and selected orbitals with their energy for compounds 2a–2c.

Code	HOMO-1	HOMO	LUMO	LUMO+1
**2a**	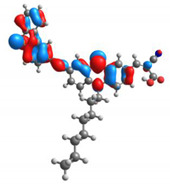	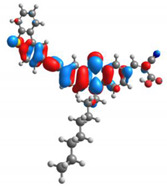	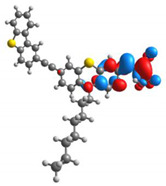	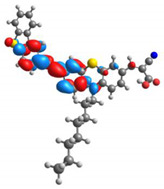
	−5.95 eV	−5.39 eV	−2.60 eV	−1.50 eV
**2b**	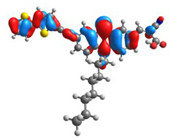	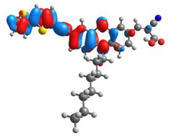	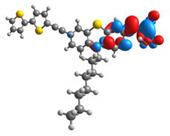	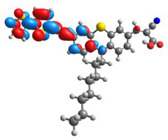
	−5.80 eV	−5.23 eV	−2.60 eV	−1.94 eV
**2c**	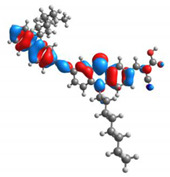	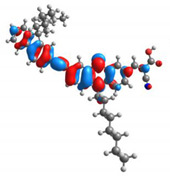	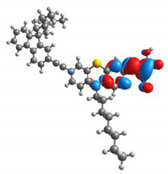	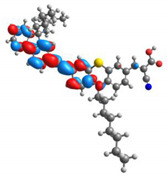
	−5.82 eV	−5.28 eV	−2.52 eV	−1.60 eV

**Table 3:** The frontier orbitals with the percentage contributions of particular parts of the molecules to their creation, energies, and values of ΔE of 2a–2c/(TiO_2_)_9_.

Code	HOMO (eV)	LUMO (eV)	ΔE (eV)
**2a/(TiO_2_)_9_**	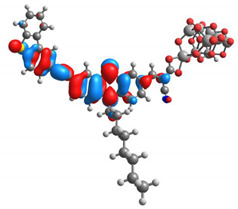	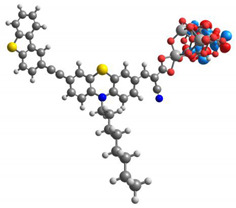	1.95
	−5.35	−3.40	
**2b/(TiO_2_)_9_**	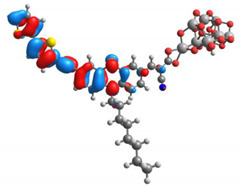	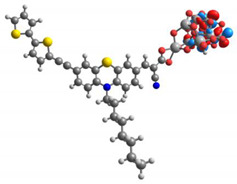	1.80
	−5.19	−3.39	
**2c/(TiO_2_)_9_**	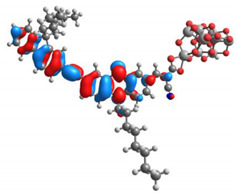	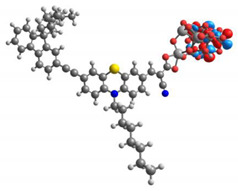	1.89
	−5.28	−3.39	

**Table 4:** Photophysical data for 2a–2c and precursors 1a–1c recorded in a chloroform solution.

Code	λ_max_ (nm) (ε (10^4^M^−1^cm^−1^))	E_g_^OPT^ (eV)
**1a**	249 (8.4), 268 (9.0), 290 (9.0), 395 (1.6)	2.63
**1b**	279 (5.8), 357 (2.6), 399 (2.0)	2.61
**1c**	296 (5.1), 307 (5.3), 325 (5.7), 395 (2.0)	2.63
**2a**	248 (2.6), 265 (2.3), 313 (2.6), 440 (0.87)	2.30
**2b**	325 (2.4), 453 (1.4)	2.25
**2c**	284 (2.1), 325 (2.4), 424 (0.63)	2.24
E_g_^OPT^ = 1240/λ		

**Table 5:** Calculated by TD-DFT, the lowest energy absorption bands with oscillator strengths compared to experimental data, values of light-harvesting efficiency (LHE) for 2a–2c.

Code	Exp (nm)	Exchange-Correlation Functional	Calculated Wavelengths (nm) (Oscillator Strengths)	Transitions	LHE
**2a**	440	CAM-B3LYP	371.80 (0.7598)	H-1->LUMO (19%), HOMO->LUMO (65%)	0.826
		wB97XD	355.93 (0.8533)	HOMO->LUMO (55%), H-1->LUMO (21%)	0.860
**2b**	452	CAM-B3LYP	378.12 (1.3308)	HOMO->LUMO (46%), H-1->LUMO (23%), HOMO->L+1 (19%)	0.953
		wB97XD	362.45 (1.5094)	HOMO->LUMO (35%), HOMO->L+1 (28%), H-1->LUMO (21%)	0.969
**2c**	423	CAM-B3LYP	383.67 (0.9218)	HOMO->LUMO (65%), H-1->LUMO (22%)	0.880
		wB97XD	373.24 (0.9951)	HOMO->LUMO (57%), H-1->LUMO (25%)	0.899

**Table 6:** The photovoltaic parameters of tested DSSC, based on dyes 2a–2c registered under AM 1.5 G illumination (100 mW/cm^2^).

Sensitizer	V_oc_ (mV)	J_sc_ (mA/cm^2^)	FF (−)	PCE (%)
**N719**	631	12.34	0.46	3.56
**2a**	700	17.96	0.48	6.22
**2b**	631	11.87	0.54	4.22
**2c**	703	12.08	0.56	4.80

## Supplementary Materials

The following are available online at https://www.mdpi.com/1996-1944/13/10/2292/s1, The detailed procedure for obtaining of molecules 1a–1c and 2a–2c, Figure S1–S12: ^1^H and ^13^C NMR spectra for 1a–1c and 2a–2c compounds, Figure S13: DSC thermograms of 2a–2c registered during I and II heating run., Figure S14: electrochemical properties of 2a–2c, Figure S15: combined IPCE spectra, Table S1: peak-to-peak separation data for 2a–2c.
