# Supplementary material for: Investigations of New Phenothiazine-Based Compounds for Dye-Sensitized Solar Cells with Theoretical Insight
Source: Materials (Basel). 2020 May 15;13(10):2292. doi: 10.3390/ma13102292 (PMC7287579; doi:10.3390/ma13102292)
Supplement: Supplementary file 1 [file materials-13-02292-s001.pdf]

materials

Supplementary Materials: Investigations of New Phenothiazine-Based Compounds for Dye-Sensitized Solar Cells with Theoretical Insight

Aneta Slodek [1,*], Dawid Zych [2], Grażyna Szafraniec-Gorol [1], Paweł Gnida [3], Marharyta Vasylieva [3] and Ewa Schab-Balcerzak [1,3,*]

[1] Institute of Chemistry, University of Silesia, Szkolna 9, 40-006 Katowice, Poland; grazyna.szafraniec-gorol@us.edu.pl
[2] Independent Researcher, Poland; dawidzych92@gmail.com
[3] Centre of Polymer and Carbon Materials, Polish Academy of Sciences, M. Curie-Sklodowska 34, 41-819 Zabrze, Poland; pgnida@cmpw-pan.edu.pl (P.G.); mvasylieva@cmpw-pan.edu.pl (M.V.)
* Correspondence: a.slodek@wp.pl or aneta.slodek@us.edu.pl (A.S.), ewa.schab-balcerzak@us.edu.pl or eschab-balcerzak@cmpw-pan.edu.pl (E.S.-B.)

Synthesis of Compounds 1a-1c

7. -[(Dibenzothiophen-2-yl)ethynyl]-10-octyl-10H-phenothiazin-3-carbaldehyde (1a)

A mixture of 7-(2-[(trimethylsilyl)ethynyl]-10-octyl-10*H*-phenothiazin-3-carbaldehyde (II) 1.4 g (2.38 mmol) and 2-bromodibenzothiophene 480 mg (1.84 mmol) dissolved in 90 mL of dry tetrahydrofuran (THF). The mixture was stirred under argon for 30 minutes. The catalytic mixture of [Pd(PPh_3)$_4$] 424 mg (0.36 mmol) and CuI 35 mg (0.36 mmol) were added and then 1M solution of tetrabutylammonium fluoride (TBAF) in THF 2.4 mL (2.4 mmol) was injected through the septum and the mixture was heated at 75 °C for 24h. After being cooled to room temperature, the reaction mixture was evaporated under reduced pressure. The crude product was purified by column chromatography using $CHCl_3$ as eluent. Yield: 40% (384 mg).

^{1}H NMR (400 MHz, $CDCl_3$) δ 9.80 (s, 1H), 8.29 (m, 1H), 8.15 (m, 1H), 7.86 (m, 1H), 7.82 (d, *J* = 8.0 Hz, 1H), 7.63 (dd, *J* = 7.6 Hz, 1.6 Hz, 1H), 7.58 (m, 2H), 7.48 (m, 2H), 7.35 (dd, *J* = 7.6 Hz, 1.6 Hz, 1H), 7.31 (s, 1H), 6.90 (d, *J* = 8.4 Hz, 1H), 6.84 (d, *J* = 8.4 Hz, 1H), 3.88 (t, *J* = 6.8 Hz, 2H), 1.81 (m, 2H), 1.26 (m, 10H), 0.88 (m, 3H). ^{13}C NMR (101 MHz, $CDCl_3$) δ 189.91, 150.02, 143.39, 139.76, 139.48, 135.70, 134.94, 131.35, 130.99, 130.25, 130.11, 129.65, 128.42, 127.16, 124.68, 124.63, 124.48, 123.93, 122.87, 122.77, 121.73, 119.31, 118.44, 115.66, 114.97, 90.07, 88.36, 48.16, 31.71, 29.18, 29.13, 26.77, 26.69, 22.61, 14.08.

HRMS (ESI): *m/z* calcd. for $C_{35}H_{31}NOS_2$: 545.1842 [M]; found 545.1840.

7. -{2-[(2,2′-bithiophen-5-yl)ethynyl]}-10-octyl-10H-phenothiazin-3-carbaldehyde (1b)

A mixture of 7-bromo-10-octyl-10*H*-phenothiazin-3-carbaldehyde (I) 2.5 g (5.97 mmol), 5-(2-[(trimethylsilyl)ethynyl]-2,2'-bithiophene 2.2 g (8.37 mmol) in 120 mL dry tetrahydrofuran (THF) was stirred under argon for 15 minutes. The catalytic mixture of [Pd(PPh_3)$_4$] 1.38 g (1.19 mmol) and CuI 0.23 g (1.19 mmol) was added and then 1M solution of tetrabutylammonium fluoride (TBAF) in THF (8.4 mL, 8.4 mmol) was injected through the septum and the mixture was heated at 65 °C for 24h. After being cooled to room temperature, the mixture was filtered and the filtrate was evaporated under reduced pressure. The crude product was purified by column chromatography using $CHCl_3$ as eluent. Yield 77% (2.43 g).

^{1}H NMR (400 MHz, $CDCl_3$) δ 9.81 (s, 1H), 7.64 (dd, *J* = 8.4, 2.0 Hz, 1H), 7.57 (d, *J* = 1.6 Hz, 1H), 7.29 (dd, *J* = 5.2, 2.4 Hz, 1H), 7.24 (dd, *J* = 4.0, 1.6 Hz, 2H), 7.19 (dd, *J* = 3.6, 0.8 Hz, 1H), 7.14 (d, *J* = 3.6 Hz, 1H), 7.06 (d, *J* = 3.6 Hz, 1H), 7.02 (dd, *J* = 5.2, 3.6 Hz, 1H), 6.90 (d, *J* = 8.6 Hz, 1H), 6.82 (d, *J* = 8.6 Hz, 1H), 3.91 – 3.85 (m, 2H), 1.86 – 1.75 (m, 2H), 1.33 – 1.23 (m, 8H), 0.90 – 0.83 (m, 3H). ^{13}C NMR (101 MHz, $CDCl_3$) δ 189.88, 149.89, 143.53, 138.88, 136.74, 132.65, 131.38, 130.81, 130.11, 129.95, 128.40, 127.97, 125.01, 124.39, 124.23, 123.93, 123.57, 121.89, 117.94, 115.64, 114.99, 93.15, 83.18, 48.16, 31.72, 29.18, 29.12, 26.76, 26.66, 22.62, 14.10.

HRMS (ESI): *m/z* calcd. for $C_{31}H_{30}NOS_3$: 528.1484 [MH^+]; found 528.1483.

7. -[2-(9,9′-dibutylfluoren-2-yl)ethynyl]-10-octyl-10H-phenothiazin-3-carbaldehyde (1c)

Compound 1c was prepared from 7-bromo-10-octyl-10*H*-phenothiazin-3-carbaldehyde (**I**) (500 mg, 1.2 mmol) and 670 mg (1.79 mmol) 2-(2-trimethylsillilethynyl)-9,9′-dibutylofluorene according to the synthetic route described for 1b. Compound 1c was purified by column chromatography using hexane/dichloromethane (10:3), and then dichloromethane. Yield: 63% (482 mg).

^{1}H NMR (400 MHz, $CDCl_3$) δ 9.81 (s, 1H), 7.67 (m, 3H), 7.58 (d, *J* = 1.6 Hz, 1H), 7.51 – 7.47 (m, 2H), 7.38 – 7.29 (m, 5H), 6.90 (d, *J* = 8.4 Hz, 1H), 6.83 (d, *J* = 8.4 Hz, 1H), 3.89 (t, *J* = 6.8 Hz, 2H), 1.98 (t, *J* = 8.3 Hz, 4H), 1.88 – 1.77 (m, 2H), 1.44 (m, 2H), 1.28 (m, 8H), 1.09 (m, 4H), 0.90 – 0.85 (m, 4H), 0.68 (t, *J* = 7.3 Hz, 6H), 0.60 (m, 3H). ^{13}C NMR (101 MHz, $CDCl_3$) δ 189.89, 151.03, 150.80, 150.04, 143.27, 141.46, 140.42, 131.34, 130.93, 130.53, 130.22, 130.11, 128.42, 127.54, 126.90, 125.86, 124.50, 123.88, 122.91, 121.34, 120.00, 119.65, 118.64, 115.65, 114.97, 91.06, 88.36, 55.06, 48.16, 40.20, 31.72, 29.18, 29.13, 26.77, 26.69, 25.92, 23.07, 22.62, 14.09, 13.82.

HRMS (ESI): *m/z* calcd. for $C_{44}H_{49}NOS$: 639.3535 [M]; found 639.3529.

Synthesis of Compounds 2a-2c

2. -Cyano-3-[7-(dibenzothiophen-2-yl)ethynyl]-10-octyl-10H-phenothiazin-3-yl)acrylic acid (2a)

A mixture of 7-[dibenzothiophen-2-yl)ethynyl]-10-octyl-10*H*-phenothiazin-3-carbaldehyde (1a) 0.06 g (0.11 mmol) and cyanoacetic acid 0.04 g (0.46 mmol) were dissolved in 10 mL of chloroform. The mixture was stirred under argon for 30 minutes. Then 0.1 mL of piperidine 0.087 g (1.00 mmol) was added and the mixture was heated at 60 °C for 24 h. After being cooled to room temperature, the water was added (30 mL) and the mixture was extracted with chloroform (3 times with 15 mL). The organic layers were collected and dried over anhydrous $MgSO_4$. After filtration, the solvent was removed under reduced pressure and the crude product was purified by chromatography ($CHCl_3$/MeOH, 15:1) to give a dark red solid of 2a. Yield: 52%.

^{1}H NMR (400 MHz, $CDCl_3$) δ 8.28 (s, 1H), 8.15 (s, 1H), 8.01 (s, 1H), 7.84 (m, 3H), 7.58 (m, 2H), 7.48 (m, 2H), 7.35 (d, *J* = 8.8 Hz, 1H), 7.24 (m, 1H), 6.79 (m, 2H), 3.82 (m, 2H), 1.80 (m, 2H), 1.28 (m, 10H), 0.89 (m, 3H).^{13}C NMR (101 MHz, $CDCl_3$) δ 167.57, 154.17, 149.13, 142.52, 139.73, 139.50, 135.68, 134.91, 131.84, 131.53, 131.08, 130.30, 130.10, 129.67, 127.16, 125.62, 124.69, 124.63, 123.87, 123.19, 122.85, 122.79, 121.75, 119.25, 118.70, 115.91, 115.56, 114.97, 98.02, 90.32, 88.30, 48.30, 31.73, 29.19, 29.15, 26.79, 26.57, 22.62, 14.10. Anal. Calcd for $C_{38}H_{32}N_2O_2S_2$: C 74.48, N 4.57, H 5.26; Found: C 74.12, N 4.37, H 5.13. DSC: (I run) T_m=68 °C; (II run) T_g = 64 °C.

2. -Cyano-3-(7-(2,2′-bithien-5-yl)ethynyl)-10-octyl-10H-phenothiazin-3-yl)acrylic acid (2b).

Compound 2b was prepared from 7-(2-(5,5'-bithienyl)ethynyl)-10-octyl-10*H*-phenothiazin-3-carbaldehyde (1b) (160 mg, 0.30 mmol) according to the synthetic route described for 2a. Compound 2b was purified by column chromatography ($CHCl_3$/MeOH, 20:1) afforded a dark red solid. Yield: 45%.

^{1}H NMR (400 MHz, DMSO) δ 7.98 (s, 1H), 7.82 (d, *J* = Hz, 1H), 7.73 (s, 1H), 7.58 (m, 1H), 7.49 – 7.36 (m, 2H), 7.36 – 7.25 (m, 3H), 7.17 – 7.05 (m, 3H), 3.99 – 3.87 (m, 2H), 1.68 (m, 2H), 1.35 (m, 2H), 1.21 (m, 8H), 0.82 (m, 3H).^{13}C NMR (151 MHz, $CDCl_3$) δ 166.03, 142.71, 139.25, 138.83, 136.76, 134.25, 132.66, 132.43, 131.94, 130.72, 129.66, 129.52, 128.83, 127.97, 125.88, 124.98, 124.20, 123.57, 123.18, 121.88, 114.92, 114.16, 103.19, 93.18, 83.29, 38.91, 31.75, 29.72, 29.18, 24.00, 22.99, 22.65, 14.14. Anal. Calcd for $C_{34}H_{30}N_2O_2S_3$: C 68.65, N 4.71, H 5.08; Found: C 68.38, N 4.56, H 5.01. DSC: (I run) T_m = 56 °C; (II run) T_g = 43 °C.

2. -Cyano-3-(7-[(2-(9,9′-dibutylfluoren-2-yl)ethynyl)]-10-octyl-10H-phenothiazin-3-yl)acrylic acid (2c).

Compound 2c was prepared from compound 1c (55 mg, 0.088 mmol) according to the synthetic route described for 2a. Compound 2c was purified by column chromatography ($CHCl_3$/MeOH, 20:1) afforded a dark red solid. Yield: 55%.

^{1}H NMR (400 MHz, DMSO) δ 7.90 (s, 1H), 7.85 (d, *J* = 3.6 Hz, 1H), 7.82 (d, *J* = 4.8 Hz, 1H), 7.79 (d, *J* = 8.8 Hz, 1H), 7.71 – 7.68 (m, 4H), 7.49 (d, *J* = 8.0 Hz, 1H), 7.45 (m, 1H), 7.41 (d, *J* = 8.8 Hz, 1H), 7.36 (s, 1H), 7.15 (d, *J* = 8.4 Hz, 1H), 7.09 (d, *J* = 8.8 Hz, 1H), 3.94 (m, 2H), 2.01 (m, 4H), 1.63 (m, 4H), 1.25 (m, 4H), 1.02 (m, 4H), 0.86 (m, 8H), 0.62 (m, 6H), 0.44 (m, 3H). ^{13}C NMR (151 MHz, $CDCl_3$) δ 165.99, 150.99, 150.77, 142.86, 141.38, 140.40, 134.20, 130.81, 130.60, 129.95, 129.51, 128.83, 127.50, 126.89, 126.36, 125.78, 123.24, 122.90, 121.41, 120.00, 119.67, 118.36, 118.26, 115.74, 115.20, 114.87, 114.10, 95.11, 91.04, 88.49, 55.02, 48.11, 40.22, 38.92, 31.75, 29.72, 29.68, 29.27, 29.18, 29.00, 26.85, 26.48, 25.92, 23.08, 22.64, 14.13, 13.85.Anal. Calcd for $C_{47}H_{50}N_2O_2S$: C 79.85, N 3.96, H 7.13; Found: C 79.57, N 3.74, H 7.08. DSC: (I run) T_m = 58 °C; (II run) T_g = 59 °C.

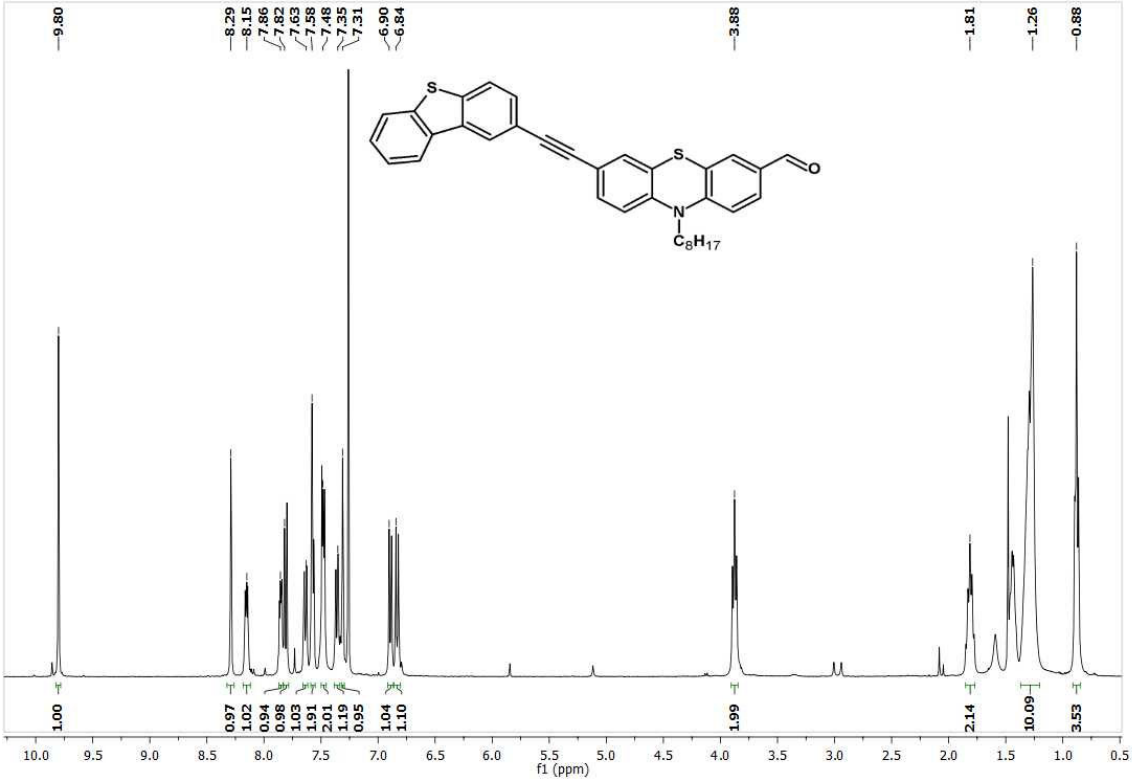

Figure S1. The ^{1}H spectrum of 1a in $CDCl_3$.

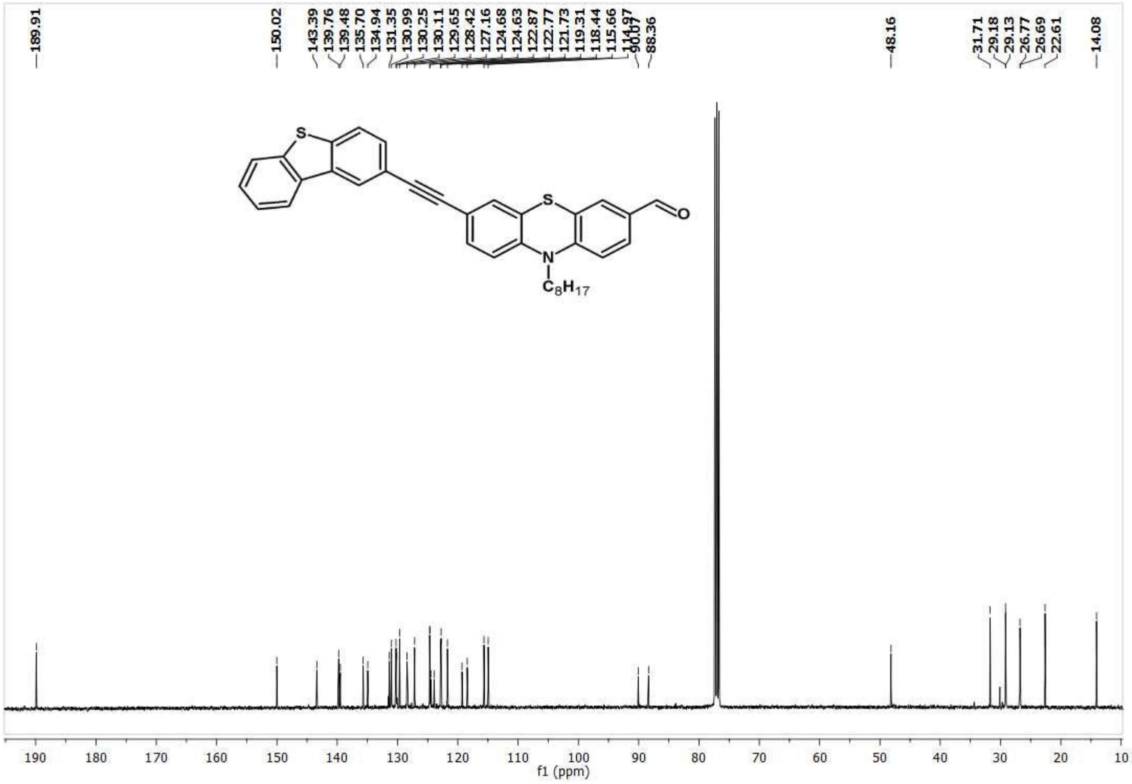

Figure S2. The ^{13}C spectrum of 1a in $CDCl_3$.

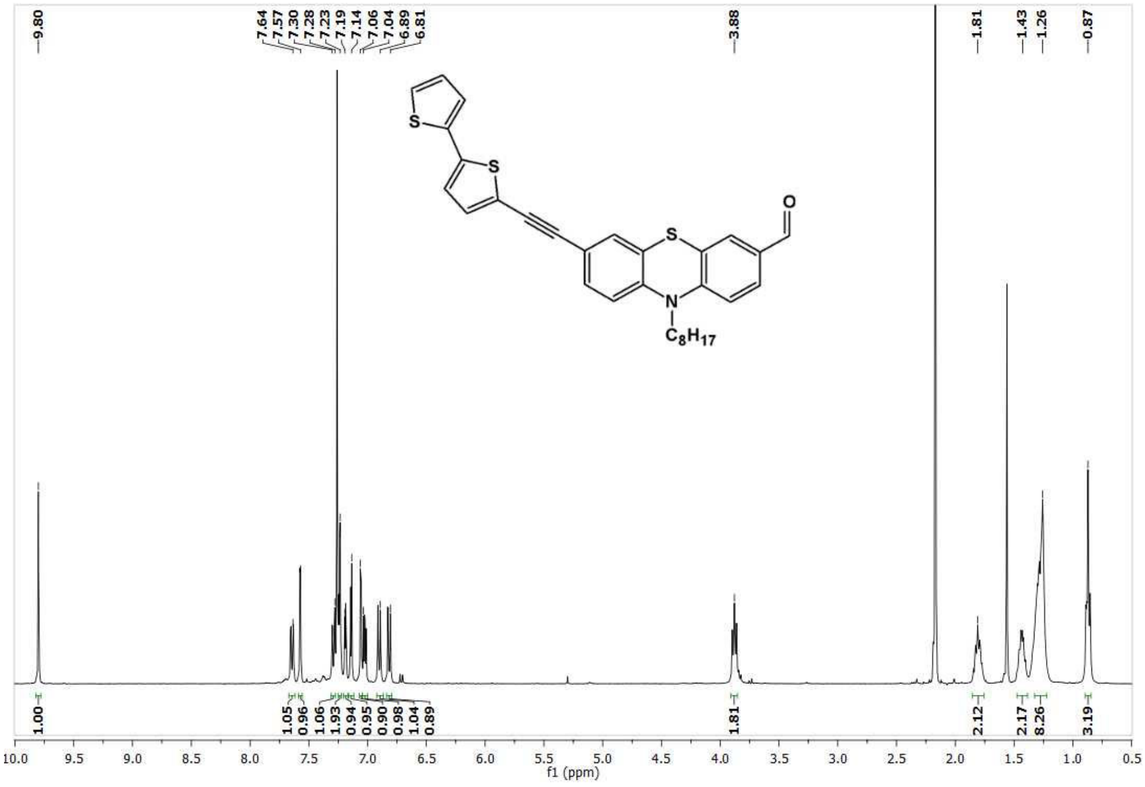

Figure S3. The ^{1}H spectrum of 1b in $CDCl_3$.

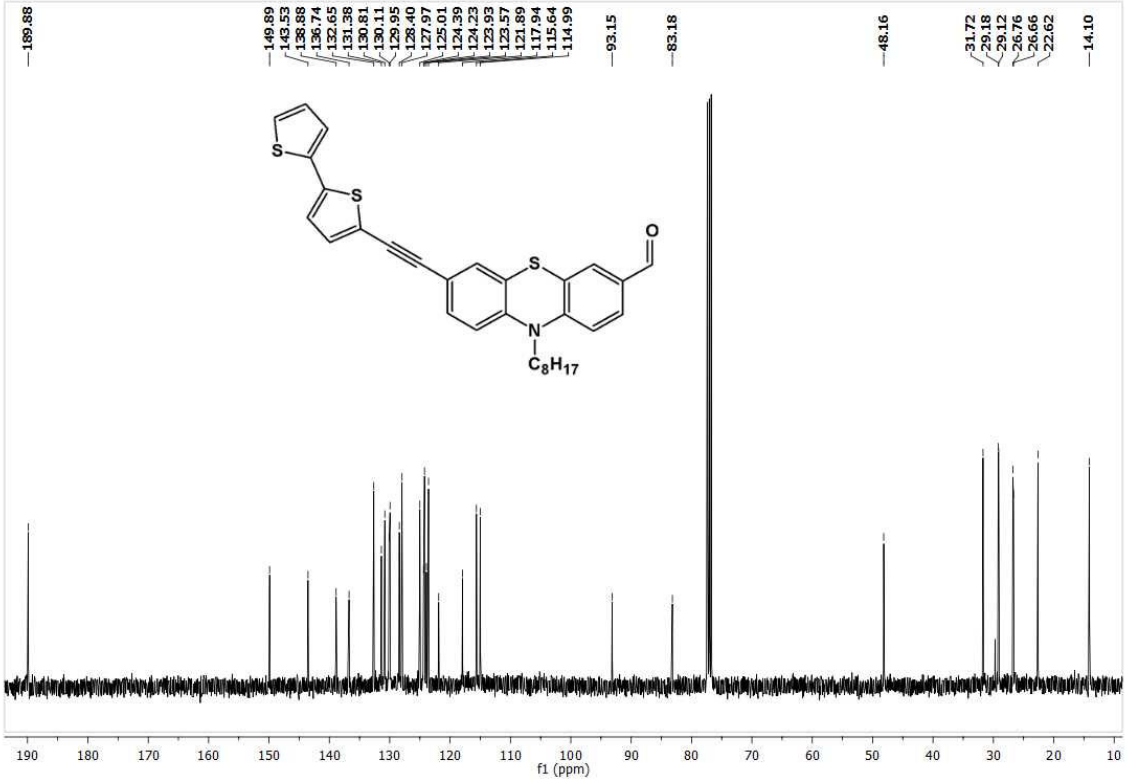

Figure S4. The ^{13}C spectrum of 1b in $CDCl_3$.

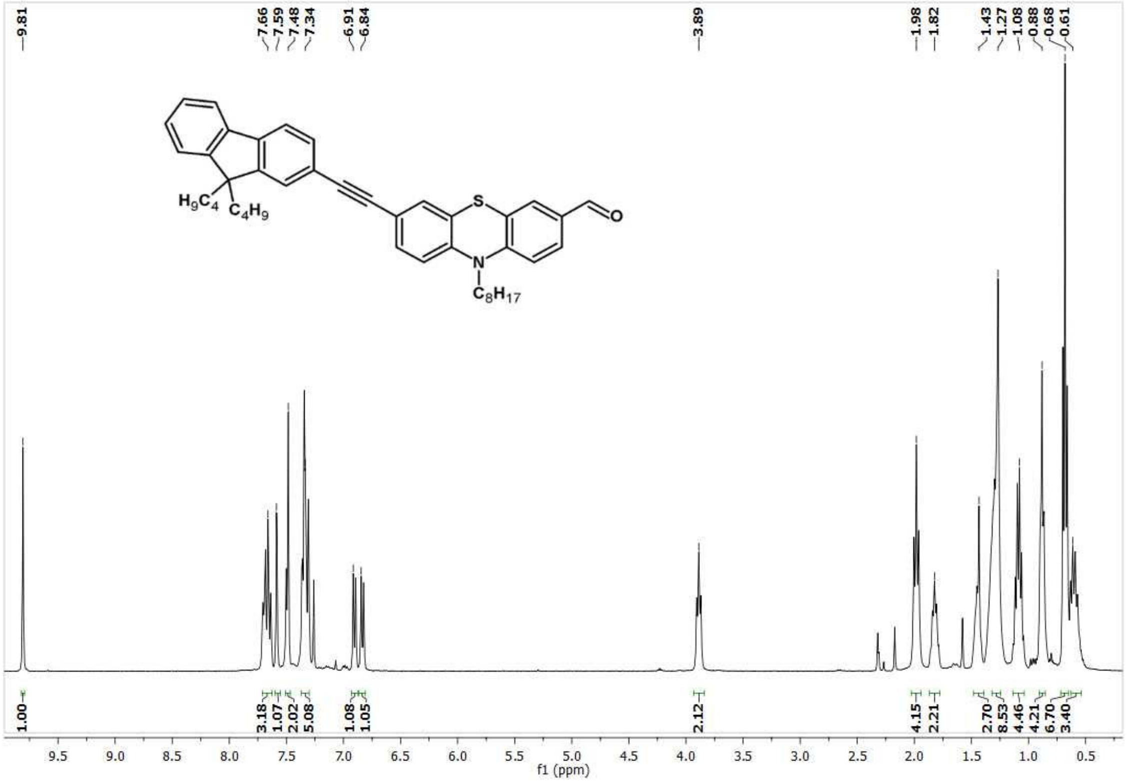

Figure S5. The ^{1}H spectrum of 1c in $CDCl_3$.

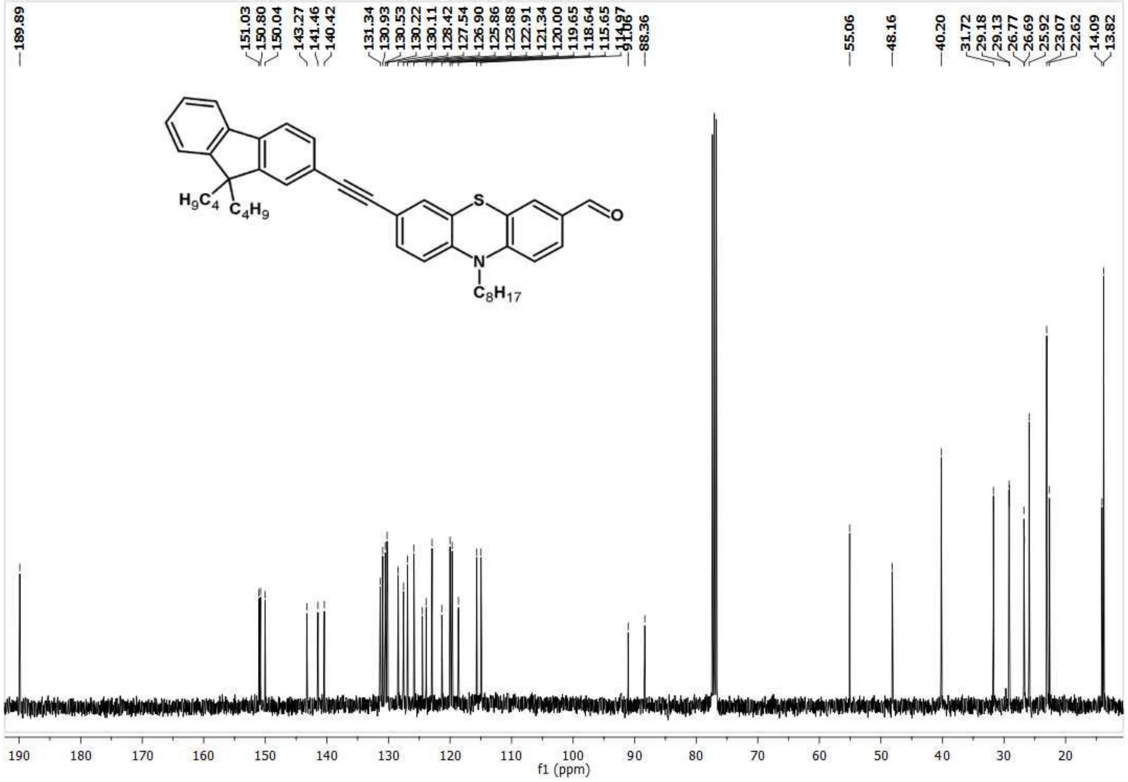

Figure S6. The ^{13}C spectrum of 1c in $CDCl_3$.

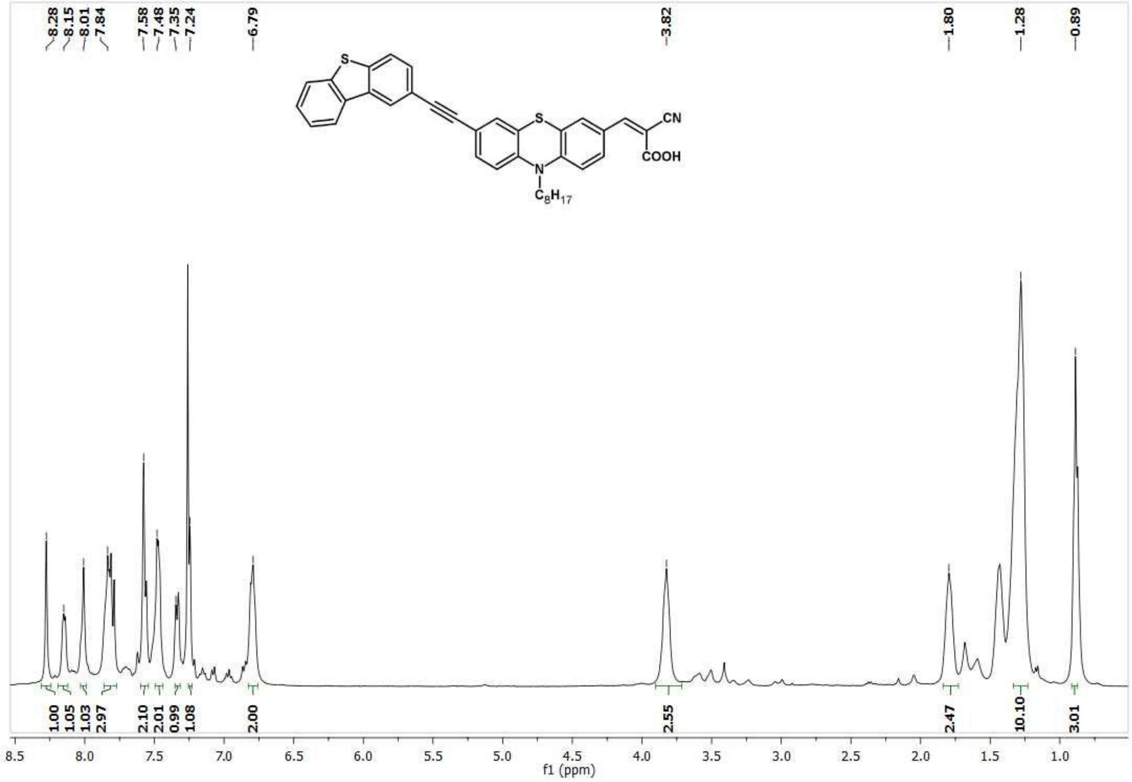

Figure S7. The ^{1}H spectrum of 2a in $CDCl_3$.

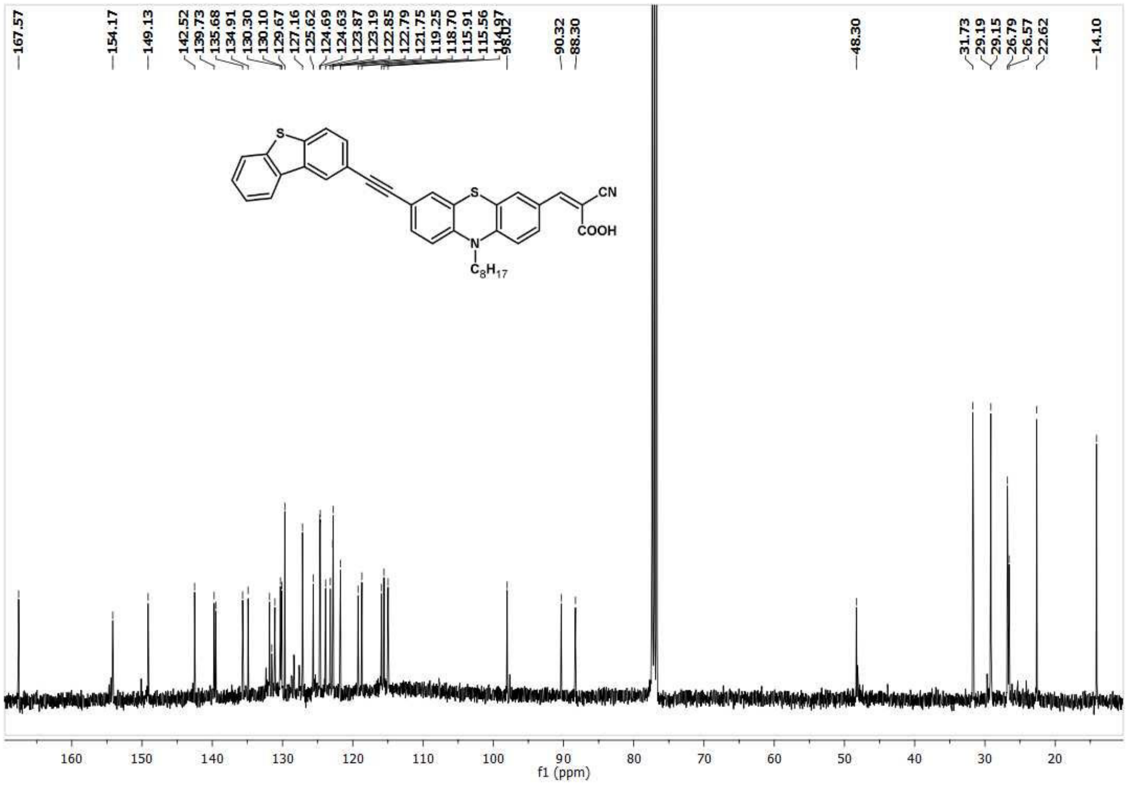

Figure S8. The ^{13}C spectrum of 2a in $CDCl_3$.

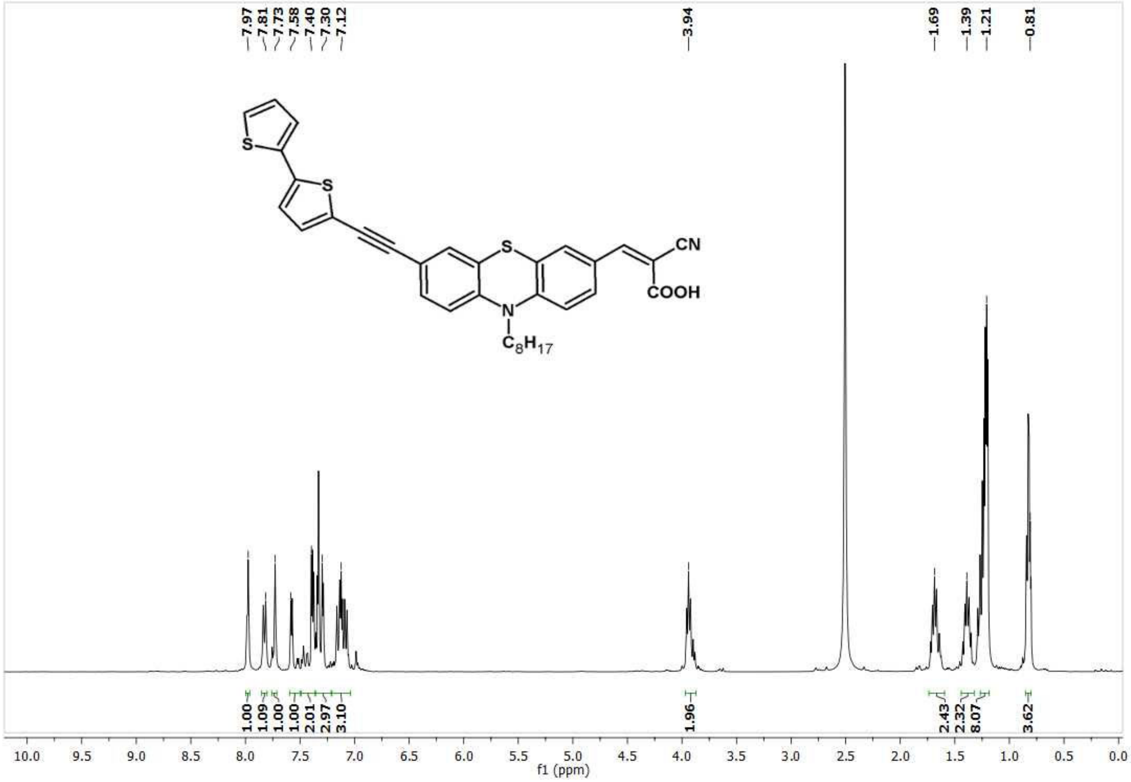

Figure S9. The ^1H spectrum of 2b in $CDCl_3$.

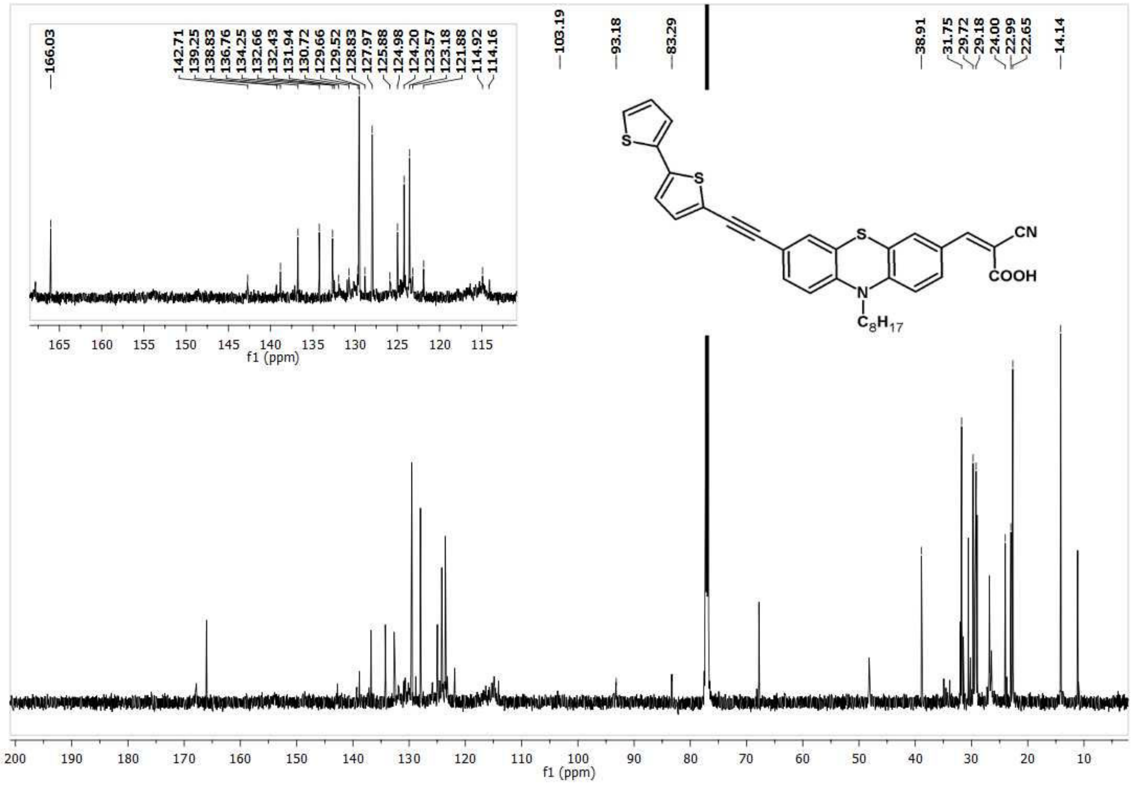

Figure S10. The ^{13}C spectrum of 2b in $CDCl_3$.

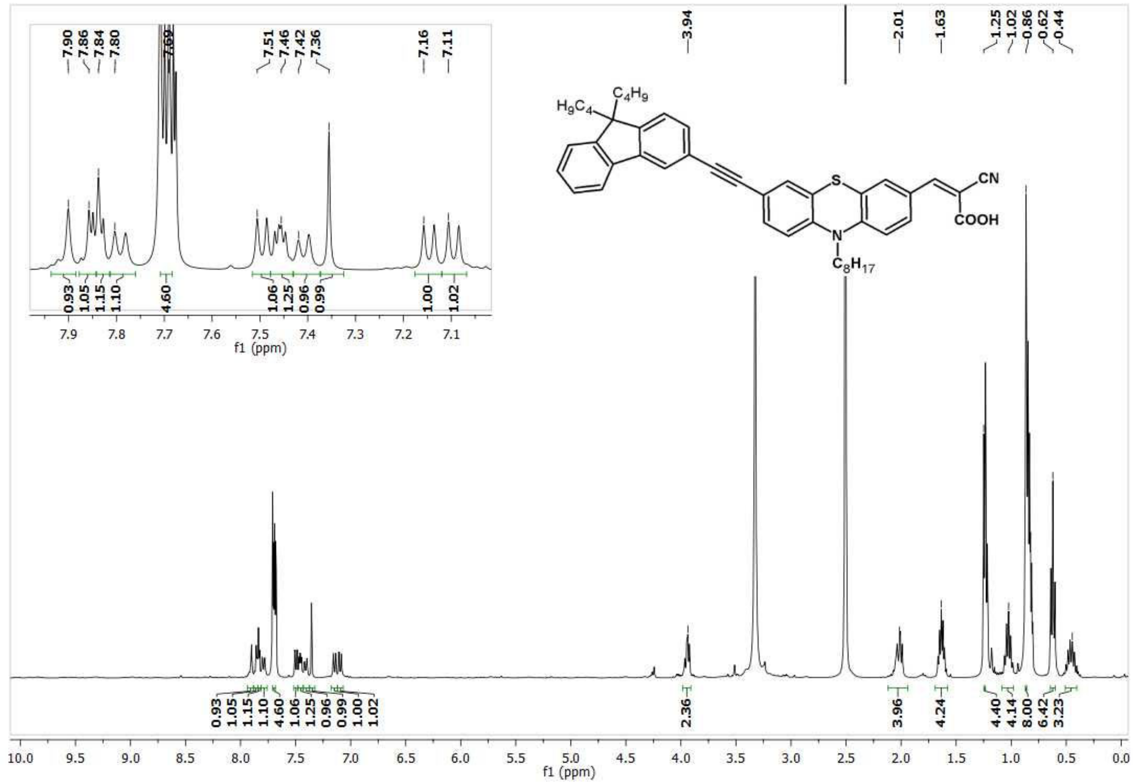

Figure S11. The ^{1}H spectrum of 2c in $CDCl_3$.

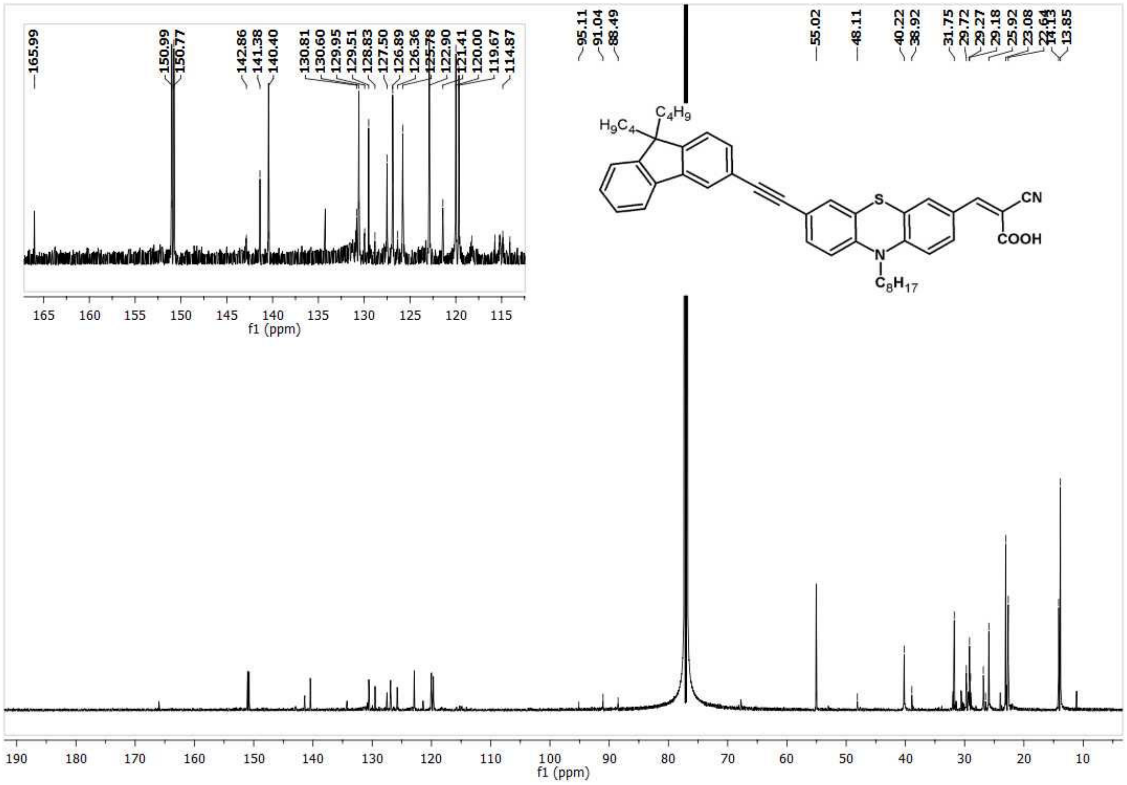

Figure S12. The ^{13}C spectrum of 2c in $CDCl_3$.

Thermal Properties

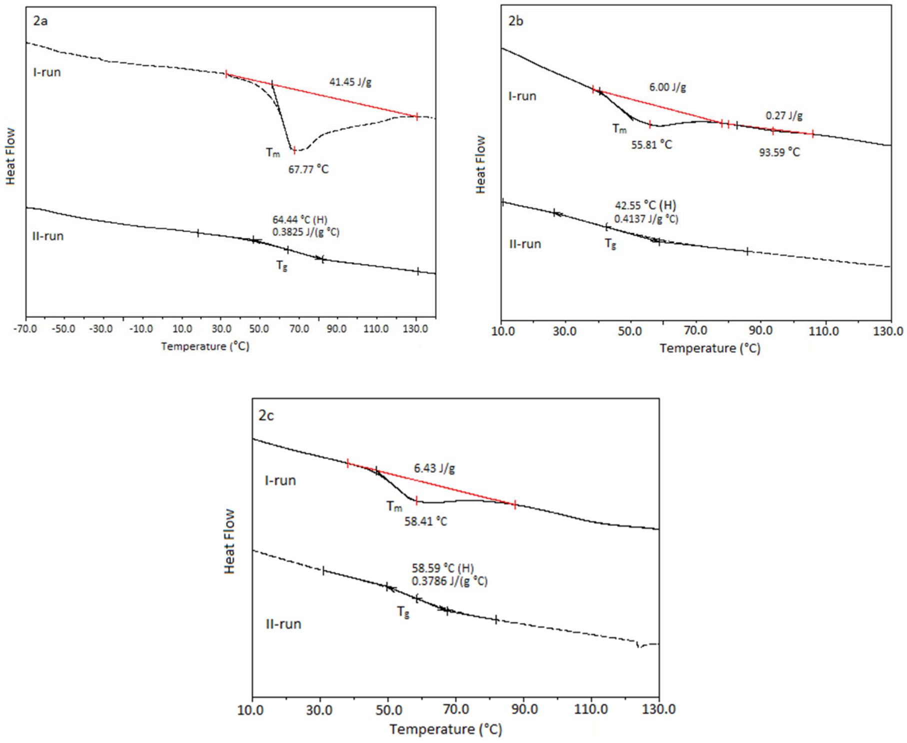

Figure S13. DSC thermograms of 2a-2c registered during the first and the second heating scan.

Electrochemical Properties

(a) **(b)**

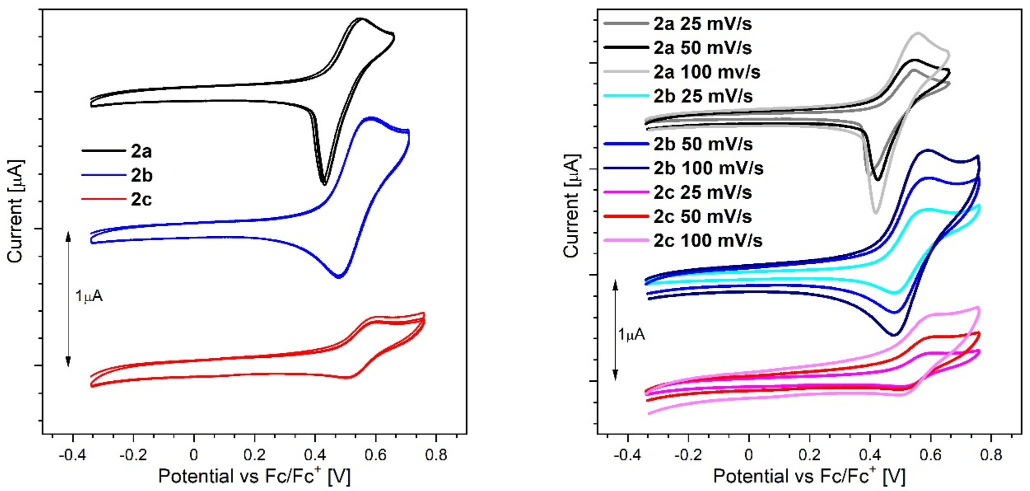

Figure S14. **(a)** CV scans of investigated compounds 2a-2c and **(b)** CV curves of the oxidation process with different scan rates.

Table S1. Peak to peak separation data of investigated compounds 2a-2c.

Code	Scan rate (mV/s)	Potential of peak separation (V)	Number of electrons
	25	0.1216	2.0610
**2a**	50	0.1241	2.1033
	100	0.1233	2.0898
	25	0.1206	2.0440
**2b**	50	0.1180	2.0000
	100	0.1192	2.0203
	25	0.1182	2.0033
**2c**	50	0.1227	2.0796
	100	0.1183	2.0508

Photovoltaic Properties

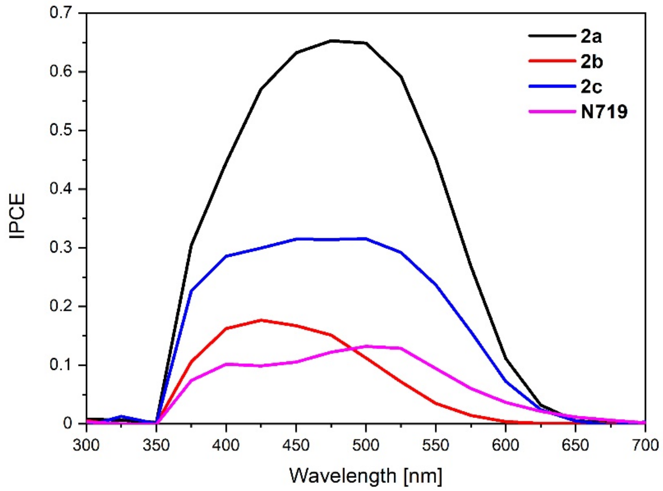

Figure S15. The combined IPCE spectra of devices containing photoanode sensitized with phenothiazine derivatives 2a-2c and N719.

© 2020 by the authors. Submitted for possible open access publication under the terms and conditions of the Creative Commons Attribution (CC BY) license (http://creativecommons.org/licenses/by/4.0/).
